# Commercial Price Variation for Common Services in General Surgery

**DOI:** 10.1001/jamanetworkopen.2025.17818

**Published:** 2025-06-25

**Authors:** Alexander P. Philips, Christopher M. Whaley

**Affiliations:** 1 Center for Advancing Health Policy Through Research (CAHPR), Brown University School of Public Health, Providence, Rhode Island; 2 Warren Alpert Medical School of Brown University, Providence, Rhode Island; 3 Department of Health Services, Policy, and Practice, Brown University School of Public Health, Providence, Rhode Island

## Abstract

This cross-sectional study uses Hospital Price Transparency and Transparency in Coverage data to examine variation in commercial insurance payment rates for common surgical services.

## Introduction

Clinicians face large variation in rates from commercial insurers.^1^ Unlike Medicare rates, commercial rates are established through a negotiation process that is not transparent, resulting in rates that are highly variable and opaque.^2^ The private market lacked meaningful price transparency for patients and purchasers until the recent implementation of Hospital Price Transparency and Transparency in Coverage (TiC) rules.

Although studies examining TiC data exist and have shown notable price variation,^3^ commercial price variation across professionals, facilities, payers, and geography for common surgical procedures has not been widely examined. Clinicians play a key role in advising patients on care decisions and can benefit from understanding the wide range of prices offered by insurers to help them guide patients in navigating cost considerations and selecting affordable care options. Additionally, the lack of transparent price information limits the ability of policymakers to appropriately design policies that ensure health care affordability. We used the TiC data to examine variation in commercial insurance payment rates for common surgical services.

## Methods

In this cross-sectional study, we examined price data from ClarifyHealth for common surgical services from 4 large national insurers (BlueCross BlueShield [BCBS], UnitedHealthcare, Cigna, and Aetna), which constitute 78% of market share.^4^ We analyzed commercial prices (excluding Medicare Advantage and Medicaid Managed Care) for practitioners and facilities with billed claims during the 2023 contract year for 10 common general surgery procedures. Prices reflect the allowed amount, which is the amount negotiated between an insurer and practitioner for a given *Current Procedural Terminology* (*CPT*) code (distinct from the chargemaster rate), and includes both payments from the insurer and patient cost-sharing responsibilities. We analyzed distributional differences in prices (mean, median, and percentiles), coefficients of variation, and price indices by payer (see the eAppendix in Supplement 1). We followed the STROBE reporting guidelines. Data analysis was performed using R statistical software version 4.4.1 (R Project for Statistical Computing). Statistical significance was determined with the *t* test and set at 2-sided *P* < .05.

## Results

For professional data, our sample had approximately 2.45 million price points and 97 740 unique physicians. For facility data, our sample included approximately 132 000 price points and 5298 facilities. The magnitude of facility prices was approximately 9 times higher than professional prices for surgical procedures and 4 times higher for endoscopic procedures (Table). Price variation was moderate for professional services and high for facility services, especially for endoscopic procedures. For example, diagnostic colonoscopy (*CPT* 45378) has a median facility cost ranging from $925 to $3571 depending on the payer. Our price indices indicate professional prices are highest for UnitedHealthcare and BCBS, and facility prices are highest for Aetna and UnitedHealthcare (Table and eAppendix in Supplement 1).

**Table.** Prices and Price Variation for Common Surgical and Endoscopic Services^a^

Service code and description	Spending, $			
	Professional		Facility	
	Mean (SD)	Median (IQR)	Mean (SD)	Median (IQR)
43235, Esophagogastroduodenoscopy with brush/wash	506 (269)	425 (344-558)	2519 (1714)	2075 (1192-3396)
43239, Esophagogastroduodenoscopy with biopsy	642 (336)	544 (434-714)	2597 (1822)	2124 (1160-3505)
44970, Laparoscopic appendectomy	1081 (544)	920 (705-1268)	10 427 (5555)	9000 (6454-12 945)
45378, Diagnostic colonoscopy	580 (330)	469 (383-646)	2529 (1796)	2014 (1135-3404)
45380, Colonoscopy with biopsy	734 (406)	607 (492-801)	2944 (1989)	2388 (1436-3923)
45385, Colonoscopy with lesion removal	783 (441)	645 (515-870)	2926 (1979)	2382 (1437-3895)
47562, Laparoscopic cholecystectomy	1240 (622)	1056 (815-1447)	10 037 (5330)	8696 (6223-12 446)
47563, Laparoscopic cholecystectomy with imaging	1317 (634)	1135 (874-1550)	10 345 (5600)	8915 (6355-12 844)
49650, Laparoscopic inguinal hernia– initial	780 (409)	659 (509-903)	10 025 (5375)	8619 (6211-12 390)
49651, Laparoscopic inguinal hernia–recurrent	991 (508)	845 (656-1138)	10 443 (5689)	9078 (6365-13 033)
Price index by payer source				
All 4 payers	1		1	
Aetna	0.81		1.33	
BlueCross BlueShield	1.02		0.86	
Cigna	0.84		0.55	
UnitedHealthcare	1.15		1.02	

^a^ These data represent October 2024 TiC data from ClarifyHealth, which aggregates price data for the 2023 contract year. Prices reflect the negotiated rate, which is the amount transferred from insurer to practitioner or facility for a given *Current Procedural Terminology* code (distinct from the chargemaster rate). Price indices are constructed by calculating the weighted average ratio of each insurer’s procedure-specific price to the mean price across all insurers, with weights adjusted to reflect spending differences. This results in a single metric where an index of 1.0 represents the national average price, while values below or above 1.0 indicate lower or higher prices respectively. Additional details on price index construction are found in the eAppendix in Supplement 1.

As a descriptive example of price variation for surgical services, the Figure maps the variation in prices for diagnostic colonoscopies. Professional prices have an upper limit of approximately 2 times its lower bound, and facility prices have an upper limit of approximately 3 times its lower bound. Similar findings were present for other procedures.

**Figure.**
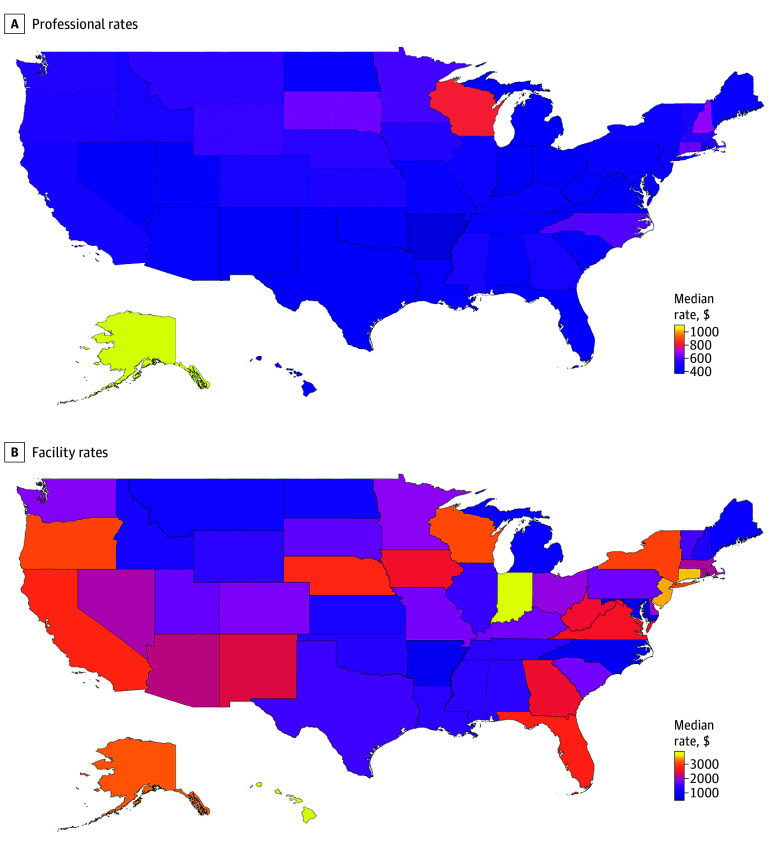
Map of Median Rates by State for Diagnostic Colonoscopy Maps show professional rates (A) and facility rates (B) for diagnostic colonoscopy (*Current Procedural Terminology* code 45378).

## Discussion

The findings of this cross-sectional study highlight substantial variation in commercial pricing for general surgery services, with facility prices exhibiting greater variability, especially for endoscopic procedures. The geographic variation in prices further underscores the influence of local market dynamics, such as hospital consolidation, competition, and regional cost structures, on health care pricing. Outlier states (eg, Wisconsin and Alaska) with higher than average professional prices may be explained by strong physician practices (ie, those with greater market leverage in negotiating with insurers, typically achieved through consolidation, specialized expertise, or regional dominance)^2^ or Medicare payment policy.^5^ A limitation of this study is that we cannot track how much of the difference in payments translates to differences in physician compensation.

Future research should examine how surgical prices vary across different care settings and how site-neutral payment policies might affect commercial market prices. Future work may also examine the underlying causes of price variation in surgical care. If price variation reflects clinical or perceived quality variation, purchasers and policymakers must find the balance between receiving higher-quality care and spending financial resources elsewhere. Other studies have indicated minimal associations between prices and care quality or efficiency.^6^ If price variation is instead driven by consolidation or anticompetitive contracting, then regulators should design policies that ensure competitive health care markets.

## Notes

Supplement 1. **eAppendix. **Supplemental Methods
eReferences

Supplement 2.
Data Sharing Statement
