# Supplementary material for: Commercial Price Variation for Common Services in General Surgery
Source: JAMA Netw Open. 2025 Jun 25;8(6):e2517818. doi: 10.1001/jamanetworkopen.2025.17818 (PMC12199050; doi:10.1001/jamanetworkopen.2025.17818)
Supplement: Supplement 1. — eAppendix. Supplemental Methods eReferences [file jamanetwopen-e2517818-s001.pdf]

Supplemental Online Content

Philips AP, Whaley C. Commercial price variation for common services in general surgery. *JAMA Netw Open*. 2025;8(6):e2517818. doi:10.1001/jamanetworkopen.2025.17818

eAppendix. Supplemental Methods

eReferences

This supplemental material has been provided by the authors to give readers additional information about their work.

© 2025 Philips AP et al. *JAMA Network Open*.

eAppendix: Supplemental Methods

A: Price Index Construction

The Transparency-in-Coverage (TiC) data contain detailed information on insurer- and provider-specific negotiated rates. To make these rates interpretable and single-dimensional, we construct a price index that aggregates the full set of weights into a single metric. This single metric allows for a more straightforward comparison of rates across insurers and geographies.

To construct this price index metric, we use existing approaches that have constructed similar price measures using medical claims data (e.g. Dunn, Shapiro, and Liebman 2013; Neprash et al. 2015). We define procedure-specific weights (w) for CPT code c as:

$$w_c = \frac{price_c * q_c}{\sum_{c=1}^{C=10}(price_c * q_c)}$$

These weights represent the aggregate share of spending accounted for each procedure code (c). Intuitively, each procedure's weight represents the spending on that code as the annual share of spending across the total ten selected procedures. To construct total spending (price times quantity (q)), for both each individual procedure code and across all 10 codes, we use the commercial claim volume data from Clarify (described in Appendix B).

Next, for each insurer b, we calculate the price index as:

$$index_b = \sum_{b=1}^{B=4} \frac{price_{cb}}{price_c} * w_c$$

This index is the weighted average ratio of each procedure's insurer-specific price ($price_{cb}$) to the mean price for each procedure across all insurers $price_c$. The weights (w_c) calculated earlier adjust for differences in total spending (e.g., procedures that account for a larger portion of total spending are adjusted upwards). Intuitively, this price index measures the deviations from the national average price, after accounting for differences in procedure spending. An index of 1.0 represents that the insurer's price is at the average, while an index below 1.0 represents lower-priced procedures, and above 1.0 represents higher-priced procedures.

B: Transparency in Coverage (TiC) Data

In this paper, we analyze 2023 "Transparency-in-Coverage" (TiC) data on insurer prices. The 2020 TiC Executive Order mandates insurance companies to publish negotiated rates for all commercially insured contracts, providing procedure- and provider-specific price lists for all insurers.

Despite being released in July 2021, these data have seen limited use due to their structural complexity. However, recent research confirms high concordance between TiC data, hospital pricing data, and Marketscan commercial claims.[1,2] One study demonstrated that insurer-disclosed prices in TiC data have a 0.975 correlation coefficient compared to hospital-disclosed prices, with 77.4% of prices matching exactly and 84.4% falling within 10% variance.[2]

Recently, a small number of private entities specializing in Transparency in Coverage have arisen to aggregate and clean this data, providing it to the business community as well as academic researchers. To

analyze the TiC data, we use data from one such third-party source, Clarify Health, under a data use agreement.

We obtained TiC data from Clarify Health, a third-party aggregator, under a data use agreement. Clarify Health compiles TiC data from monthly insurer postings and addresses the challenge of "zombie rates" (listed prices for providers unlikely to perform specific procedures).[3] To overcome this limitation, Clarify links TiC data with a complete sample of Medicare fee-for-service enrollees and commercial insurance claims data, covering approximately 270 million people.

To ensure data quality, we:

- Restricted observations to TiC prices for providers with documented claims for relevant service codes

- Removed outliers by including only prices between 75% and 1000% of Medicare rates

- Limited our sample to the four national insurers (Blue Cross Blue Shield, UnitedHealth Group, Cigna, and Aetna), which represent 78% of market share[4]

For our analysis, we used weighted average negotiated prices across all plan types (PPO, HMO) as calculated by Clarify Health. This means if an insurer has enrollees in both PPO and HMO plans with different pricing structures, our analysis incorporates a weighted average of these prices.

C: IRB Statement

Brown University Institutional Review Board deemed this cross-sectional study exempt from ethics review and informed consent because it was not human participant research.

eReferences:

1. Wang Y, Meiselbach M, Anderson GF, Bai G. Hospital Pricing Information Consistent Between Transparency-In-Coverage Data And Other Commercial Data Sources. doi:10.1377/forefront.20231108.269718

2. Henderson M, Mouslim M. Cross-Validation of Insurer and Hospital Price Transparency Data. 2024;30:e247-e250.

3. Oakes AH, Ikard M, Patton C, et al. Understanding Variation in Negotiated Rates Using Novel Health Plan Price Transparency Data. *JAMA Health Forum*. 2024;5(9):e243020. doi:10.1001/jamahealthforum.2024.3020

4. Guardado J, Kane C. *Competition in Health Insurance: A Comprehensive Study of U.S. Markets*. American Medical Association; 2023.

© 2025 Philips AP et al. *JAMA Network Open*.
