# Supplementary material for: Commercial Price Variation for Common Services in General Surgery
Source: JAMA Netw Open. 2025 Jun 25;8(6):e2517818. doi: 10.1001/jamanetworkopen.2025.17818 (PMC12199050; doi:10.1001/jamanetworkopen.2025.17818)
Supplement: Supplement 2. — Data Sharing Statement [file jamanetwopen-e2517818-s002.pdf]

Data Sharing Statement

Philips. Commercial Price Variation for Common Services in General Surgery. *JAMA Netw Open*. Published June 27, 2025. doi:10.1001/jamanetworkopen.2025.17818

Data
Data available: No

Additional Information
Explanation for why data not available: Per our data use agreement with ClarifyHealth, we are unable to provide the raw data for analysis upon request.
